# *N*-acylhomoserine lactonase-based hybrid nanoflowers: a novel and practical strategy to control plant bacterial diseases

**DOI:** 10.1186/s12951-022-01557-9

**Published:** 2022-07-26

**Authors:** Yan Chen, Pengfu Liu, Jiequn Wu, Wanqing Yan, Saixue Xie, Xuanrong Sun, Bang-Ce Ye, Xiaohe Chu

**Affiliations:** Collaborative Innovation Center of Yangtze River Delta Region Green Pharmaceuticals, Zhejiang University of Technology, Hangzhou, 310014 Zhejiang China

**Keywords:** Biocontrol, Quorum sensing and quenching, AHL lactonases, Enzyme-based hybrid nanoflower, *Erwinia carotovora*, *Burkholderia glumae*, Robustness

## Abstract

**Background:**

The disease caused by plant pathogenic bacteria in the production, transportation, and storage of many crops has brought huge losses to agricultural production. *N*-acylhomoserine lactonases (AHLases) can quench quorum-sensing (QS) by hydrolyzing acylhomoserine lactones (AHLs), which makes them the promising candidates for controlling infections of QS-dependent pathogenic bacteria. Although many AHLases have been isolated and considered as a potentially effective preventive and therapeutic agents for bacterial diseases, the intrinsically poor ambient stability has seriously restricted its application.

**Results:**

Herein, we showed that a spheroid enzyme-based hybrid nanoflower (EHNF), AhlX@Ni_3_(PO_4_)_2_, can be easily synthesized, and it exhibited 10 times AHL (3OC8-HSL) degradation activity than that with free AhlX (a thermostable AHL lactonase). In addition, it showed intriguing stability even at the working concentration, and retained ~ 100% activity after incubation at room temperature (25 °C) for 40 days and approximately 80% activity after incubation at 60 °C for 48 h. Furthermore, it exhibited better organic solvent tolerance and long-term stability in a complicated ecological environment than that of AhlX. To reduce the cost and streamline production processes, CSA@Ni_3_(PO_4_)_2_, which was assembled from the crude supernatants of AhlX and Ni_3_(PO_4_)_2_, was synthesized. Both AhlX@Ni_3_(PO_4_)_2_ and CSA@Ni_3_(PO_4_)_2_ efficiently attenuated pathogenic bacterial infection.

**Conclusions:**

In this study, we have developed *N*-acylhomoserine lactonase-based hybrid nanoflowers as a novel and efficient biocontrol reagent with significant control effect, outstanding environmental adaptability and tolerance. It was expected to overcome the bottlenecks of poor stability and limited environmental tolerance that have existed for over two decades and pioneered the practical application of EHNFs in the field of biological control.

**Graphical Abstract:**

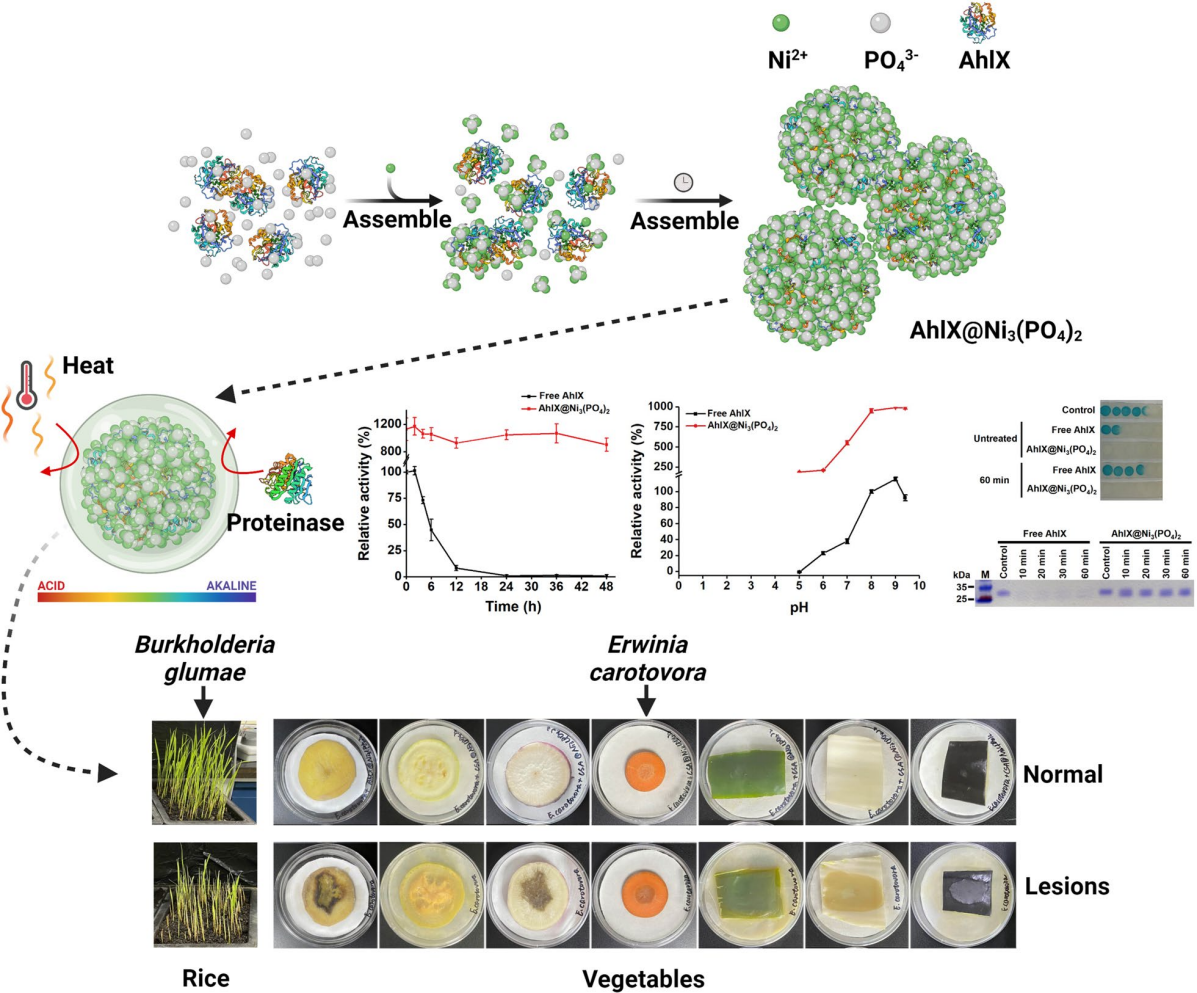

**Supplementary Information:**

The online version contains supplementary material available at 10.1186/s12951-022-01557-9.

## Introduction

Plant bacterial diseases severely decrease the global crop yield each year. Treating plant bacterial diseases with chemical bactericides is effective but causes many environmental problems [1, 2]. The pathogenicity of many Gram-negative bacteria is coordinated by the quorum sensing (QS) system and is based on diffusible *N*-acylhomoserine lactones (AHLs) [3]. Soft rot from crops, such as potato, cabbage, radish, garlic, onion, cucumber, carrot, eggplant, squash, and tomato, is caused by *Erwinia carotovora* due to the production of exoenzymes (protease, cellulases and pectinases) in response to 3-oxo-hexanoyl-L-homoserine lactone [4, 5]. Seedling and grain rot from rice caused by *Burkholderia glumae* result from toxoflavin biosynthesis, lipase production and secretion, as well as motility, in response to *N*-octanoyl homoserine lactone [6–8]. Quorum quenching (QQ) enzymes, which can interfere with the infection process (which is modulated by the QS system of bacterial pathogens) by degrading AHLs, have become promising candidates for controlling plant bacterial disease in the past 20 years [9–11].

According to the different mechanisms of action on AHLs, QQ enzymes are conventionally divided into lactonases, acylases, and oxidoreductases [12, 13]. Among them, AHL lactonases (AHLases) have received increasing attention due to their widespread presence [14–18]. A large group of AHLases with a broad substrate spectrum and excellent catalytic performance have been isolated and characterized. However, most of these AHLases are easily destroyed by harsh environmental elements, including proteases, high temperatures, and high salt levels, which strongly limits their application in controlling plant bacterial diseases. To address stability issues, some thermostable AHLases, such as GKL (an orthologous phosphotriesterase-like lactonase from the thermophile *Geobacillus kaustophilus*) [19], AiiA_AI96_ (a thermostable *N*-acylhomoserine lactonase from *Bacillus* sp. strain AI96) [20], Aii20J (a wide-spectrum thermostable *N*-acylhomoserine lactonase from the marine bacterium *Tenacibaculum* sp. 20 J) [21], AiiT (a thermostable *N*-acylhomoserine lactonase from the thermophilic bacterium *Thermaerobacter marianensis*) [22] and AidB (a thermostable *N*-acylhomoserine lactonase from the bacterium *Bosea* sp.) [23], were successively mined, and the thermostability of PPH (a phosphotriesterase-like lactonase) was improved by directed evolution [24]. Despite these efforts, the long-term stability of these AHLases under complicated ecological environments is not satisfactory. In the previous work, we identified a typical AHLase (called AhlX) from a marine bacterium, *Salinicola salaries* MCCC1A01339 [25]. AhlX shows highly efficient AHL degradation activity, thermostability, and salt tolerance, and can attenuate *E. carotovora* infection*.* Nevertheless, the activity of AHLase is still destroyed when it is exposed to the natural environment. It cannot be applied practically due to its inherent vulnerability in natural environments. Therefore, further improvement of AHLases stability remains quite important for their practical use.

Different from the inherent poor stability of free enzymes, enzyme immobilization based on physical, covalent, and affinity interactions with or without carrier is an effective strategy to improve enzyme stability and recyclability [26–32]. Despite this, there still exist some deficiencies in many conventional immobilization ways such as complicated preparation process, excessive cost, the use of toxic and harmful reagents, loss of activity, inferior reproducibility, and mass transfer limitations [33, 34]. Enzyme-based hybrid nanoflowers (EHNFs) are composites with a large specific surface area and porous and uniformly controllable flower-like morphology that are formed by enzymes and inorganic metal phosphates through self-assembly [35, 36]. Prepared EHNFs can effectively improve the stability, reusability and enantioselectivity of an enzyme, reduce mass transfer limitations without losing enzyme activity, and even increase the enzyme activity in many cases [37–44]. In 2012, Ge et al. accidentally discovered that enzymes can spontaneously form nanoflower-like complexes with Cu_3_(PO_4_)_2_ crystals and increase their enzyme activity by 650% [45]. Since then, an increasing number of enzymes, peptides, amino acids, nucleic acids, bioextracts, organic molecules, and metal ions have been attempted in the preparation of hybrid nanoflowers [32, 46–48]. The production process of EHNFs is convenient, fast, and does not use toxic, harmful, and expensive chemical reagents. For these reasons, EHNFs have become a highly important enzyme immobilization method and have been used in the fields of biosensors, biocatalysts, biomedicine, and wastewater treatment [39, 45, 49–52].

**Fig. 1:**
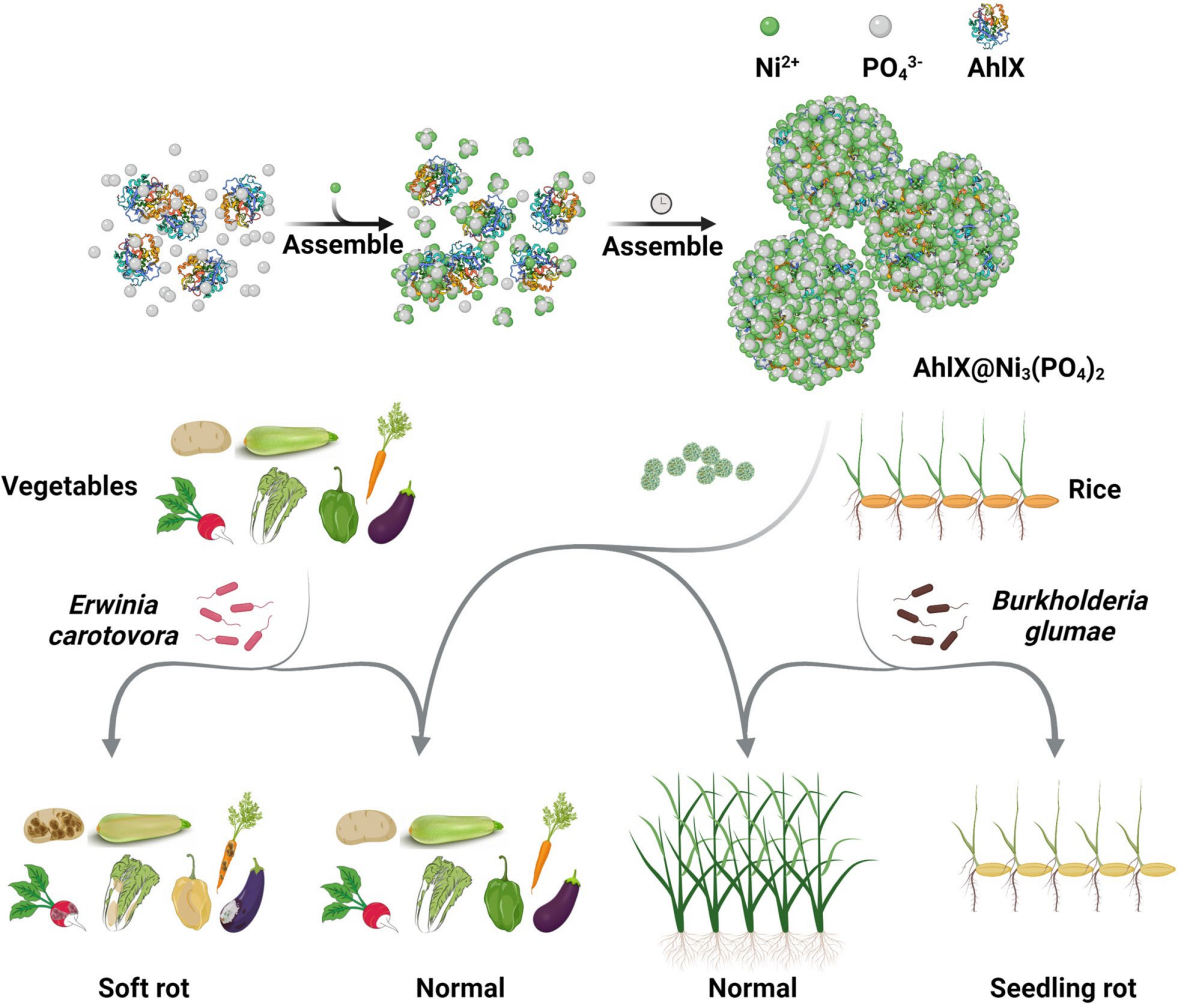
Schematic illustration of the preparation and application of AhlX@Ni_3_(PO_4_)_2_

In this study, we developed an efficient strategy to increase the activity and robustness of AHLases for breaking their limitation in practical application. AhlX-based EHNFs were synthesized and applied to quench AHL-dependent quorum sensing of phytopathogenic bacteria. As shown in Fig. 1, we prepared and characterized a highly stable and highly active AHLase EHNF, AhlX@Ni_3_(PO_4_)_2_. AhlX was first incubated with Ni^2+^ and PO_4_^3−^ and then self-assembled into an EHNF, AhlX@Ni_3_(PO_4_)_2_. AhlX@Ni_3_(PO_4_)_2_ showed an excellent thermal stability, storage stability, organic solvent tolerance and high catalytic activity. It also displayed excellent stability in a natural water environment and exhibited long-term inhibition of plant bacterial infection. The AhlX@Ni_3_(PO_4_)_2_-dependent QQ strategy should largely benefit the practical control of plant bacterial diseases.

## Materials and methods

### Bacterial strains and chemicals

*Escherichia coli* BL21 (DE3) was used to achieve a heterologous expression of the AhlX protein in the pET28a (+) vector. The *Agrobacterium tumefaciens* NT1 strain expressing AHL-regulated LacZ was used to evaluate AHL activity with the bioassay method described by Dong et al. [9]. *Erwinia carotovora* SCG1 was provided by Prof. Ziduo Liu (HuaZhong Agricultural University, Wuhan, China) and was used to test the quenching activity of AhlX@Ni_3_(PO_4_)_2_ for potato soft rot. *Burkholderia glumae* CCTCC AB 2,016,346 was purchased from the China Center for Type Culture Collection (CCTCC) and was used to test the quenching activity of AhlX@Ni_3_(PO_4_)_2_ for rice seedling rot. *N*-(3-oxooctanoyl)-l-homoserine lactone (3OC8-HSL) was purchased from Sigma Aldrich (Missouri, USA). Other chemicals, if not specified, were purchased from Sinopharm Group Co. Ltd. (Shanghai, China).

### Expression and purification of AhlX

The expression and purification of AhlX were performed as described in a previous study [25]. To assess the purity of the purified AhlX, 15% (v/v) sodium dodecyl sulfate-denatured polyacrylamide gel electrophoresis (SDS–PAGE) analysis was performed. Protein concentrations were measured with the Modified Bradford Protein Assay Kit (Sangon Biotech).

### Synthesis of AhlX-EHNFs

According to the promoting effect of divalent metal ions on free AhlX activity, nickel sulfate hexahydrate, zinc sulphate heptahydrate, manganese sulfate monohydrate, magnesium sulfate, and cobalt chloride hexahydrate were chosen to synthesize AhlX-EHNFs. The general synthetic process is as follows. Eighty microliters of aqueous divalent metal ion solution (200 mM) were added to 1 mL of 10 mM phosphate buffer (PB) with different pH values containing AhlX at different concentrations, followed by incubation at 4 °C or 25 °C for different times. For the divalent metal ion selection experiments, the pH value of PB was 8.0, the final AhlX concentration was 3.2 μM, and the incubation time was 36 h. For the pH preparation experiments, the pH range tested was from pH 5 to pH 9.4, the final AhlX concentration was 3.2 μM, and the incubation time was 36 h. For the experiments preparing the AhlX concentrations, the pH value of PB was 8.0, the final AhlX concentration ranged from 3.2 μM to 25.6 μM, and the incubation time was 36 h. For the incubation time experiments, the pH value of PB was 8.0, the final AhlX concentration was 3.2 μM, and the incubation time ranged from 0.5 h to 10 h. After that, the mixtures were centrifuged for 2 min at 12,000 rpm, the supernatants were decanted, and the precipitates were washed three times with ultrapure water. Protein concentrations in all supernatants were determined by a Modified Bradford Protein Assay Kit (Sangon Biotech). Amount of immobilized AhlX is calculated by deducting total AhlX used for immobilization from the amount of un-immobilized AhlX. And the encapsulation efficiency was defined as the ratio of AhlX immobilized to the total amount of AhlX used. The final precipitates were resuspended in 10 mM PB (pH 8.0) to a final AhlX concentration of 3.2 μM.

### Activity and kinetics assay of AhlX and AhlX-EHNFs

The activity and kinetics assay of free AhlX and AhlX-EHNFs was performed as described in a previous study [25]. Briefly, 5 µL of free AhlX or AhlX-EHNFs was added to 200 µL of 10 mM phosphate buffer (PB, pH 8.0) containing 1.6 mM 3OC8-HSL substrate and was incubated at 30 °C for 10 min. The reaction was terminated with SDS solution at a final concentration of 5%, and the remaining 3OC8-HSL was detected by high-performance liquid chromatography (HPLC) on a Diamonsil^®^C18 column (4.6 × 250 mm, 5 µm, Dikma, Beijing, China) with a mobile phase of C2H3N:HCOOH:H2O (50: 0.2: 49.8) at 1 mL min^−1^. The detection wavelength was set to 201 nm. The concentration of 3OC8-HSL was set as 0.25–10 μM during kinetic detection. Kinetic parameters were obtained by nonlinear regression analysis of the Michaelis–Menten equation with origin 2017 (Origin Laboratory.). All samples were analyzed in triplicate.

### Characterization of AhlX@Ni_3_(PO_4_)_2_ EHNF

The centrifuged AhlX@Ni_3_(PO_4_)_2_ pellet was washed three times with ultrapure water, filtered on a 0.22 µm MCE filter membrane (Millipore), and dried at room temperature for subsequent tests. The morphology and elemental analysis of AhlX@Ni_3_(PO_4_)_2_ were observed by a field emission scanning electron microscopy (SEM) S-4700 (Hitachi). The samples were coated with a thin layer of gold before they were placed in the SEM chamber. Fourier transform infrared spectroscopy (FTIR) spectra were collected on a Nicolet iS10 FTIR spectrometer (Nicolet). The conventional KBr pressed pellet method was used to prepare the samples. Each sample was scanned from 4000 to 400 cm^−1^. X-ray photoelectron spectroscopy (XPS) was performed on an Axis Ultra DLD with a monochromic Al X-ray source (Kratos). circular dichroism (CD) spectroscopy was performed with a J-815 CD spectropolarimeter (Jasco).

### Biochemical characterization of free AhlX and AhlX@Ni_3_(PO_4_)_2_

The optimum temperature and pH for free AhlX and AhlX@Ni_3_(PO_4_)_2_ activity toward 3-OC8-HSL was assessed at different temperatures (10 to 60 ℃, the activity of free AhlX at 30 ℃ was defined as 100%) and different pH values (PB for pH 5.0–9.4, the activity of free AhlX at pH 8.0 was defined as 100%) under standard conditions. To test the organic solvent tolerance of free AhlX and AhlX@Ni_3_(PO_4_)_2_, 5% of different organic solvents (ethanol, acetonitrile, isopropanol, *n*-butanol and isoamyl alcohol) were added to the reaction system. The activity of free AhlX in the absence of organic solvent was defined as 100%. The temperature stability of free AhlX and AhlX@Ni_3_(PO_4_)_2_ was measured by a preprocessing treatment at 0 to 100 ℃ for 30 min. In addition, to measure the time-dependent thermal stability of free AhlX and AhlX@Ni_3_(PO_4_)_2_, their residual activities were tested after incubation at 25℃ for 0–40 days and 60℃ for 0–48 h. All samples were analyzed in triplicate.

To detection the free AhlX and AhlX@Ni_3_(PO_4_)_2_ stabilities in the ecological environment, they were added to sterilized and unsterilized river water (in the Zhejiang University of Technology sections of the Beijing-Hangzhou Grand Canal) to a final AhlX concentration of 3.2 µM and were incubated at room temperature for 0–30 days. Mixtures were taken at day 0, day 4, day 8, day 12, day 20, and day 30 for the activity assay with the agar strip diffusion method and 15% (v/v) SDS–PAGE analysis. For proteinase K treatment, 0.3 U of proteinase K was added to 20 µL of 3.2 µM free AhlX or AhlX-EHNF solution (10 mM PB, pH 8.0) and incubated at 37 °C for 0–60 min. Next, the activity was analyzed by the agar strip diffusion method, and the remaining AhlX was detected with 15% (v/v) SDS–PAGE analysis. Before 15% (v/v) SDS–PAGE analysis, 5 µL of 0.1 M HCl solution was added into 20 µL of AhlX@Ni_3_(PO_4_)_2_ samples to release AhlX from it. All samples were analyzed in triplicate.

The agar strip diffusion method was performed in the following manner. Five microliters of the mixtures and 4 µL of 20 µM 3OC8-HSL were added to 11 µL of 10 mM PB (pH 8.0) and incubated at 30 °C for 10 min. The reaction was terminated with SDS solution at a final concentration of 5%. Ten microliters of reaction solution was dot coated on the anterior end of agar strips surface coated with *Agrobacterium tumefaciens* NT1. Cultures were grown overnight (20 h) at 30 °C, and the activity was assessed by the length of blue plaque.

### Quenching the *E. carotovora* and *B. glumae* Infection by AhlX@Ni_3_(PO_4_)_2_ and CAS@Ni_3_(PO_4_)_2_

To assess the in vitro biocontrol effect of free AhlX and AhlX@Ni_3_(PO_4_)_2_ on the soft rot caused by *E. carotovora*, 1 µL of *E. carotovora* overnight cultures (3.0 × 10^7^ CFU mL^−1^) and 10 µL of free AhlX or AhlX@Ni_3_(PO_4_)_2_ (AhlX concentration was 3.2 µM) were mixed well and inoculated in the center of the potato slices. As a control, 10 µL of ddH_2_O, 10 µL of AhlX, 10 µL of AhlX@Ni_3_(PO_4_)_2_, and 1 µL of *E. carotovora* were inoculated in the center of the potato slices. All samples were analyzed in triplicate. The progression of lesion development was photographed every 2 days after incubation at 30 °C for 0–10 days, and the lesion area was quantified using Photoshop software (Adobe).

To investigate the in vitro suppressive efficacy of CSA@Ni_3_(PO_4_)_2_ in *E. carotovora* infection, surface-sterilized potato, marrow squash, radish, carrot, pepper, Chinese cabbage, and eggplant were inoculated separately with PB (pH = 8.0, as a control), *E. carotovora*, *E. carotovora* and CSA, *E. carotovora* and CSA@Ni_3_(PO_4_)_2_. Potato, marrow squash, and pepper slices were inoculated with 11 µL of PB (pH 8.0), a mixture of 1 µL of *E. carotovora* (3.0 × 10^7^ CFU·mL^−1^) and 10 µL of PB, a mixture of 1 µL of *E. carotovora* (3.0 × 10^7^ CFU mL^−1^) and 10 µL of CSA (3.2 µM), and a mixture of 1 µL of *E. carotovora* (3.0 × 10^7^ CFU·mL^−1^) and 10 µL of CSA@Ni_3_(PO_4_)_2_ (3.2 µM of CSA) and incubated at 30 °C for 5, 5, and 3 days, respectively. Radish, carrot, and Chinese cabbage slices were inoculated with 11 µL of PB, a mixture of 1 µL of *E. carotovora* (1.5 × 10^8^ CFU·mL^−1^) and 10 µL of PB, a mixture of 1 µL of *E. carotovora* (1.5 × 10^8^ CFU·mL^−1^) and 10 µL of CSA (3.2 µM), and a mixture of 1 µL of *E. carotovora* (1.5 × 10^8^ CFU mL^−1^) and 10 µL of CSA@Ni_3_(PO_4_)_2_ (3.2 µM of CSA) and incubated at 30 °C for 3, 3, and 2 days, respectively. Eggplant slices were inoculated with 11 µL of PB, a mixture of 1 µL of *E. carotovora* (1.5 × 10^9^ CFU·mL^−1^) and 10 µL of PB, a mixture of 1 µL of *E. carotovora* (1.5 × 10^9^ CFU·mL^−1^) and 10 µL of CSA (3.2 µM), and a mixture of 1 µL of *E. carotovora* (1.5 × 10^9^ CFU·mL^−1^) and 10 µL of CSA@Ni_3_(PO_4_)_2_ (3.2 µM of CSA) and incubated at 30 °C for 2 days. To evaluate the in vivo inhibition efficacy of CSA@Ni_3_(PO_4_)_2_ in *B. glumae* infection, rice seedlings (two weeks old) were cut to the bottom 2 cm aboveground and drenched with tap water (as a control), *B. glumae* (1.0 × 10^8^ CFU·mL^−1^), *B. glumae* and CSA (3.2 µM), *B. glumae* and CSA@Ni_3_(PO_4_)_2_ (3.2 µM CSA). The infection degree was evaluated 2 weeks after treatment.

## Results and discussion

### Preparation of AhlX-EHNFs

The formation of EHNFs goes through three major steps [31, 46, 53]: (a) during nucleation, divalent metal ions react with phosphate ions (PO_4_^3−^) to form primary metallic phosphate crystals, (b) in step of growth, primary crystals combine with enzymes via coordination interaction between metal ions and carboxyl, amine and imidazole groups of enzymes to create agglomerates, and (c) in the final step, flower-like structures are eventually formed by anisotropic growth. In this study, AhlX-EHNFs were produced through the self-assembly of AhlX and metal phosphates. The influence of the metal ion kinds, temperature, pH value, enzyme concentration, and preparation time of AhlX-EHNFs was investigated.

**Fig. 2:**
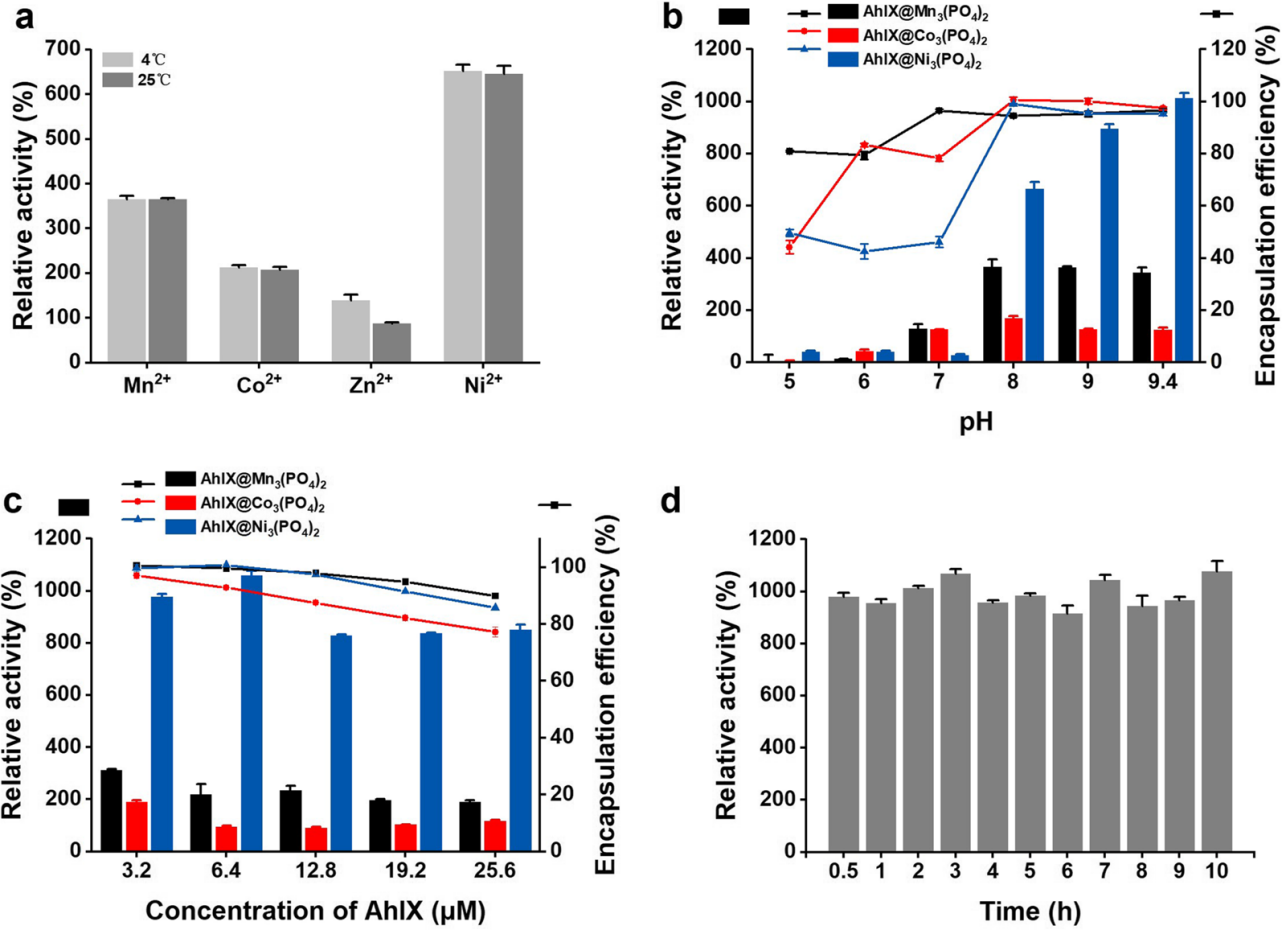
Optimization of biomineralized AhlX preparation conditions. **a** Effects of preparation temperature on the activity of biomineralized AhlX. **b** Effects of preparation pH on the activity of biomineralized AhlX. **c** Effects of AhlX concentration on the activity of biomineralized AhlX, **c** Effects of preparation time on the activity of biomineralized AhlX. The activity of free AhlX under the condition of pH 8.0 was used as reference and defined as 100%

The active center of the AhlX monomer contains a conserved Zn^2+^-binding domain, HXHXDH [23]. This site binds two Zn^2+^ ions and plays an important role in the catalytic ability of this type of enzyme [54–56]. In previous experiments, we found that Cu^2+^, Fe^2+^, Ca^2+^ possessed varying degrees of inhibition on the catalytic ability of AhlX, while five divalent metal ions, including Ni^2+^, Zn^2+^, Mg^2+^, Mn^2+^, and Co^2+^, have different promoting effects (between 1.5 and 3.5 times) on it [25]. Therefore, these five divalent metal ions were selected as the metal ion donors for the preparation of AhlX-EHNFs. The results showed that, except for the Mg^2+^ group, the other 4 groups all formed precipitation of AhlX-EHNFs (Fig. 2a). Zn^2+^ was also discarded in the subsequent experiments due to its weak influence in the activity of AhlX-EHNF. AhlX-EHNFs prepared by the ions of Co^2+^, Mn^2+^, Ni^2+^ show 2-, 3.5- and 6.5-times catalytic activity to free enzyme, respectively. No significant difference on activity of AhlX-EHNFs prepared at 4 °C and 25 °C had been found. The preparation temperature at about 25 °C was more energy-efficient and convenient.

The acidic environment was not conducive to the formation of active AhlX-EHNFs, and the protein encapsulation efficiency was higher than 95% under alkaline conditions (Fig. 2b). The concentration of PO_4_^3−^ decreased from pH 9.4 to pH 5.0 gradually. In acidic environment, PO_4_^3−^ with low concentration could not form enough metallic phosphates crystals, crystal deficiency adversely affected nucleation, agglomeration and anisotropic growth of AhlX-EHNFs, which resulted in the lower catalytic activity and protein encapsulation efficiency. Considering both catalytic activity and encapsulation efficiency, the optimum pH for preparing AhlX@Mn_3_(PO_4_)_2_, AhlX@Co_3_(PO_4_)_2_ and AhlX@Ni_3_(PO_4_)_2_ was 8.0, 8.0 and 9.4 respectively.

As the protein concentration increased from 3.2 to 25.6 µM, the encapsulation efficiency gradually decreased (Fig. 2c). The AhlX@Ni_3_(PO_4_)_2_ prepared at the protein concentration of 6.4 μM showed the best catalytic activity. The influence of preparation time from 0.5 to 10 h on the activity of AhlX@Ni_3_(PO_4_)_2_ was studied. Different from EHNFs of the most enzymes, AhlX@Ni_3_(PO_4_)_2_ with total catalytic activity could be prepared within only 0.5 h (Fig. 2d).

Finally, an optimized AhlX@Ni_3_(PO_4_)_2_, in which the activity was 10 times higher than that of free AhlX, was successfully prepared in 10 mM PB (pH = 9.4) containing 6.4 μM AhlX and 15 mM NiSO_4_ at room temperature (RT, 25 °C) for 0.5 h (Fig. 2).

### Structure characterization of AhlX@Ni_3_(PO_4_)_2_

**Fig. 3:**
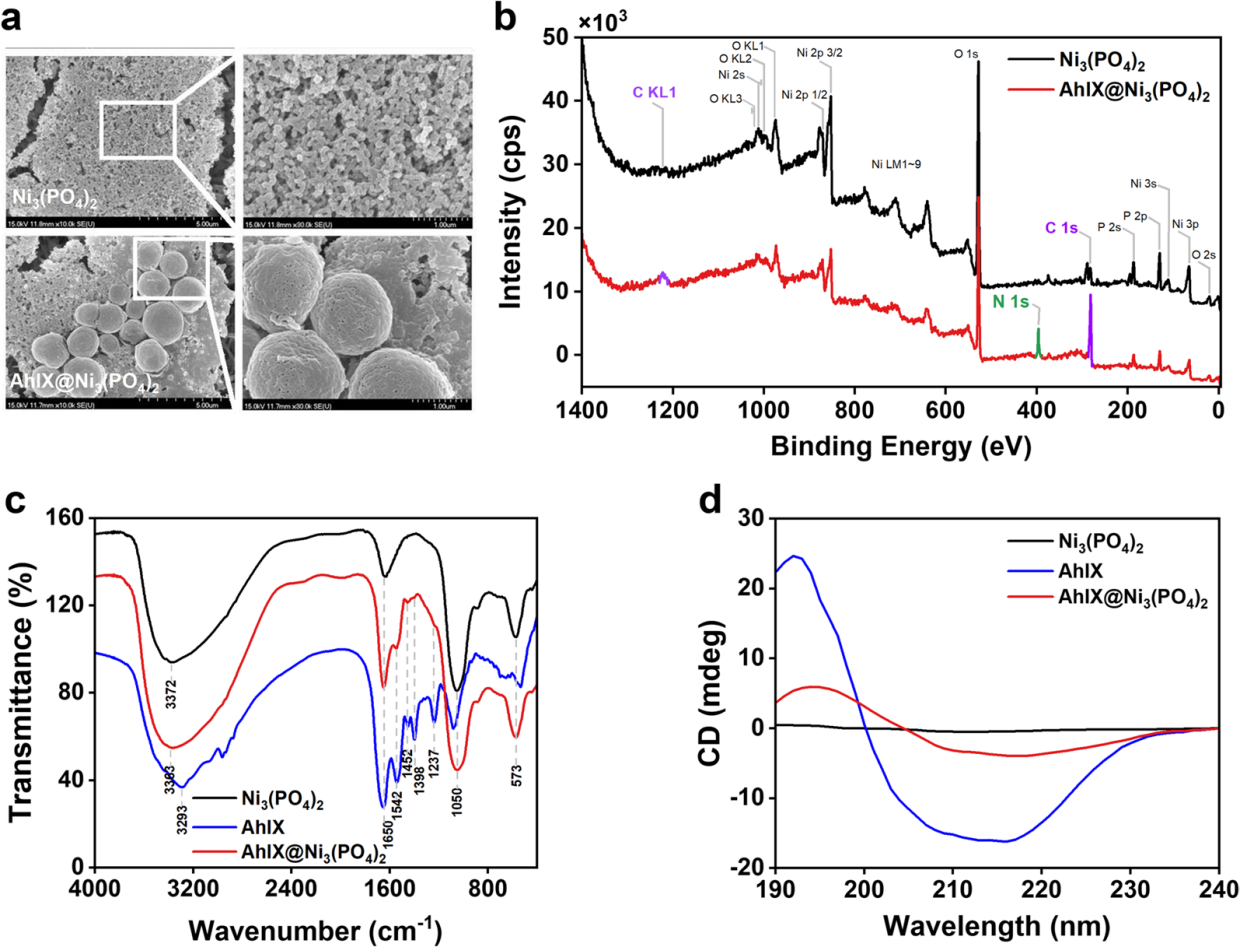
Structural characterization of the biomineralized enzyme AhlX@Ni_3_(PO_4_)_2_. **a** SEM image of the biomineralized enzyme AhlX@Ni_3_(PO_4_)_2_. **b** XPS spectra of Ni_3_(PO_4_)_2_ and AhlX@Ni_3_(PO_4_)_2_. **c** FTIR spectra of Ni_3_(PO_4_)_2_, AhlX, and AhlX@Ni_3_(PO_4_)_2_. **d** CD spectra of Ni_3_(PO_4_)_2_, AhlX, and AhlX@Ni_3_(PO_4_)_2_

To explore the morphological and structural characteristics, AhlX@Ni_3_(PO_4_)_2_ was analyzed by SEM, XPS, FTIR, and CD. It was found that AhlX@Ni_3_(PO_4_)_2_ has a spherical structure with a porous surface (average size, ~ 2 μm) and high surface-to-volume ratios, while Ni_3_(PO_4_)_2_ crystals exhibit small balls (average size, ~ 0.1 μm) and cannot form a three-dimensional stacked structure (Fig. 3a). In the presence of AhlX, AhlX can be used as a “glue” to bond small Ni_3_(PO_4_)_2_ spherical particles into a three-dimensional structure [57]. The sponge-like surface and interior portion of AhlX@Ni_3_(PO_4_)_2_ allow AHLs to penetrate freely and be degraded rapidly. Such a structure may be one of the reasons for the improvement in activity.

The full XPS spectra of Ni_3_(PO_4_)_2_ and AhlX@Ni_3_(PO_4_)_2_ revealed that they both had C, O, Ni and P elements on their surfaces (Fig. 3b). C on the surface of Ni_3_(PO_4_)_2_ particles was attributed to CO_2_ contamination from the air [58]. In contrast to Ni_3_(PO_4_)_2_, the peak of N 1 s only appeared in AhlX@Ni_3_(PO_4_)_2_, and the peaks of C 1 s and C KL1 were significantly enhanced. XPS results demonstrated the successful construction of AhlX@Ni_3_(PO_4_)_2_.

FTIR provides direct evidence of the interaction associated with the fabrication of AhlX@Ni_3_(PO_4_)_2_ (Fig. 3c). AhlX@Ni_3_(PO_4_)_2_ not only had a strong P-O stretching vibration absorption peak at 1050 cm^−1^ but also had bending vibrations of bridging phosphorous such as O = P-O at 573 cm^−1^ [59], which indicated the presence of phosphoric acid groups in AhlX@Ni_3_(PO_4_)_2_. In addition, the FTIR image of AhlX@Ni_3_(PO_4_)_2_ at 1650 cm^−1^, 1542 cm^−1^ and 1237 cm^−1^ also demonstrated the existence of the following protein secondary structure bands: an amide I band (1660 ~ 1640 cm^−1^, C = O stretching vibration), amide II band (1550 ~ 1530 cm^−1^, overlap of N–H bending vibration and C-N stretching vibration) and amide III band (1340 ~ 1220 cm^−1^) [60–62]. The peak at 1452 cm^−1^ was assigned to the in-plane bending vibration of the C-H and O–H bonds of the protein [62]. The peaks between 2800 and 3000 cm^−1^ correspond to the C-H bond stretching vibration peaks of -CH_2_ and -CH_3_ in AhlX [63]. Compared with free AhlX, no obvious peak shifts were found at the amide I, amide II and amide III bands, and no new absorption peaks appeared. The above results indicated that AhlX@Ni_3_(PO_4_)_2_ is formed by the self-assembly of AhlX and Ni_3_(PO_4_)_2_ through intermolecular forces and without the formation of covalent bonds.

Although no new chemical bond forms, the self-assembly of AhlX and Ni_3_(PO_4_)_2_ still changed the secondary structure of the protein. The shape of the CD spectra curve provides detailed information about the secondary structure of AhlX (Fig. 3d). The structure of the α-helix presents a positive peak at 192 nm and ‘w’-shaped negative peaks at approximately 208 and 222 nm, while a positive peak at 196 nm and a negative peak near 218 nm are indicative of β-sheets [64]. The CD spectra of AhlX exhibited a positive peak of α-helices at 192 nm and a negative peak of β-sheets at 216 nm. Because the Ni_3_(PO_4_)_2_ structure lacked chirality, there were no distinct peaks in the 190 ~ 240 nm UV region. After immobilization with Ni_3_(PO_4_)_2_, the peak intensity of AhlX was dramatically reduced in the CD spectra, and the positive peak at 192 nm was redshifted and appeared at 194 nm, indicating that the proportion of α-helices and β-sheets decreased and increased, respectively.

### Enzymatic properties of free AhlX and AhlX@Ni_3_(PO_4_)_2_

**Fig. 4:**
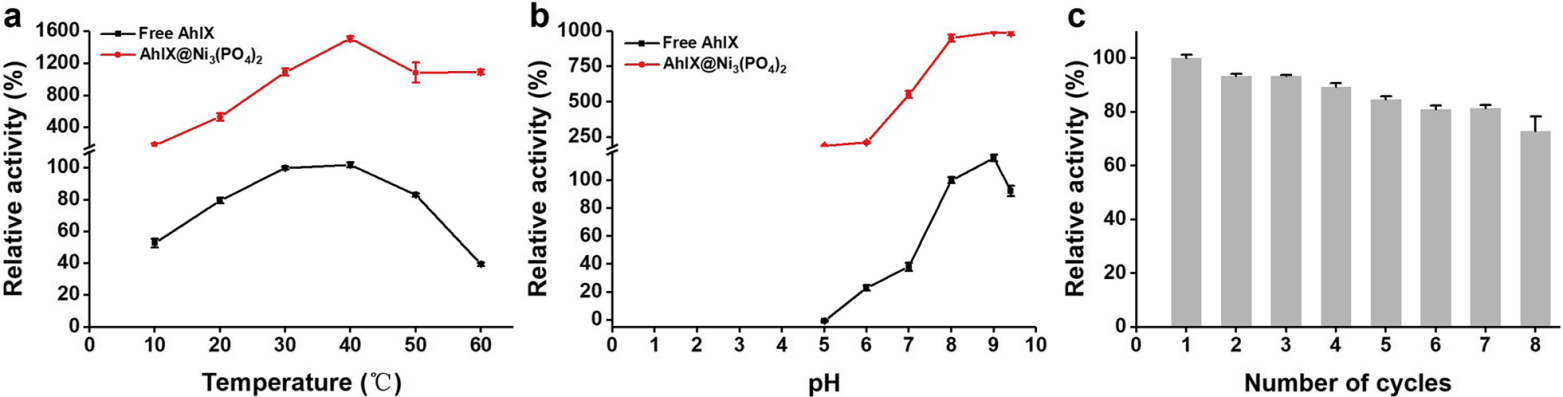
Biochemical characterization and reusability of free AhlX and AhlX@Ni_3_(PO_4_)_2_. **a** Effects of temperature on the activity of free AhlX and AhlX@Ni_3_(PO_4_)_2_. The activity of free AhlX at 30℃ was defined as 100%. **b** Effects of pH on the activity of AhlX and AhlX@Ni_3_(PO_4_)_2_. The activity of free AhlX at pH 8.0 was defined as 100%. **c** Reusability of AhlX@Ni_3_(PO_4_)_2_. The activity of AhlX@Ni_3_(PO_4_)_2_ of the first cycle was defined as 100%

The operating temperature range of AhlX@Ni_3_(PO_4_)_2_ is wider than that of free AhlX. Compared with free AhlX, AhlX@Ni_3_(PO_4_)_2_ was more resistant to high temperatures and could maintain the same activity at 30 ℃ and 60 ℃, while free AhlX lost 60% of its activity at the higher temperature (Fig. 4a). At the same time, AhlX@Ni_3_(PO_4_)_2_ had improved acid and alkali tolerance (Fig. 4b). The activity of free AhlX was completely lost at pH 5.0, while AhlX@Ni_3_(PO_4_)_2_ maintained 19.26% of its activity. There was no decreasing trend for free AhlX when the pH was greater than 9. Moreover, AhlX@Ni_3_(PO_4_)_2_ has good reusability. It is well known that free enzymes cannot be recovered after use. After immobilization, a free enzyme gains an insoluble solid-state, which allows it to be recovered after the reaction, and its usability and economy are improved. After 8 repeated uses, the remaining enzyme activity was still as high as 73% (Fig. 4c). After four cycles of use and recovery, this recovery rate was higher than 60% for the AiiA (a *N*-acylhomoserine lactonase from *Bacillus* sp. 240B1) that was immobilized onto magnetic nanoparticles [65]. As reusability was tested in a small reaction volume, inevitable physical loss during occurred with each recycling process, which increased the loss in activity. Thus, the activity was better preserved with AhlX@Ni_3_(PO_4_)_2_.

The kinetic constants *k*_cat_ and *K*_M_ of AhlX@Ni_3_(PO_4_)_2_ and free AhlX were determined (Table 1). No obvious change was found in the *K*_M_ value between free AhlX and AhlX@Ni_3_(PO_4_)_2_, indicating that the affinity of AhlX to 3OC8-HSL remained unchanged. The increase in catalytic efficiency was due to the increase in the conversion number (*k*_cat_) value. After immobilization, the *k*_cat_ value had a tenfold enhancement from 21.57 s^−1^ to 214.80 s^−1^, and the *k*_cat_/*K*_**M**_ value increased from 6.05 × 10^3^ M^−1^·s^−1^ to 6.12 × 10^4^ M^−1^·s^−1^. This result was consistent with the prior activity improvement results. The increased activity of EHNFs may be derived from the following factors [31, 45, 66]: (a) reduced mass-transfer limitations due to high surface area, (b) enzyme favorable conformation in EHNFs, and (c) synergistic effects between the enzyme molecules and the enzyme-metal ions. According to the CD results, there was an increase in the proportion of β-sheets and a decrease in the proportion of α-helices in the AhlX structure after immobilization, indicating that the hydrophobicity of AhlX increases [67, 68]. According to the study of Permyakov et al. [55], the AHLase AiiA, which belongs to MLL AHLases, degrades the Y conformation C8-C12AHL in the active state when only one water molecule is connected to Zn^2+^ in the active center. The long distance between the two Zn^2+^ allows more water molecules to approach the active center, which shifts the substrate binding mode, which is critical to the catalytic ability of AiiA. The water repellency of the active center is important for the continuous catalytic ability of AiiA. Therefore, it is not difficult to speculate that the increased hydrophobicity of AhlX@Ni_3_(PO_4_)_2_ improves the water repellency of the active center, eventually improving its catalytic ability for the Y conformation of 3OC8-HSL. In a nutshell, the increased activity of AhlX@Ni_3_(PO_4_)_2_ is mainly due to the favorable conformation of AhlX.

**Table 1:** Kinetic parameters of free AhlX and AhlX@Ni_3_(PO_4_)_2_

Kinetic parameters	AhlX	AhlX@Ni_3_(PO_4_)_2_
*K*_M_ (M)	3.56 × 10^–3^	3.51 × 10^–3^
*k*cat (s-1)	21.57	214.80
*k*cat/*K*M (M-1·s-1)	6.05 × 10^3^	6.12 × 10^4^

### Stability of Free AhlX and AhlX@Ni_3_(PO_4_)_2_

**Fig. 5:**
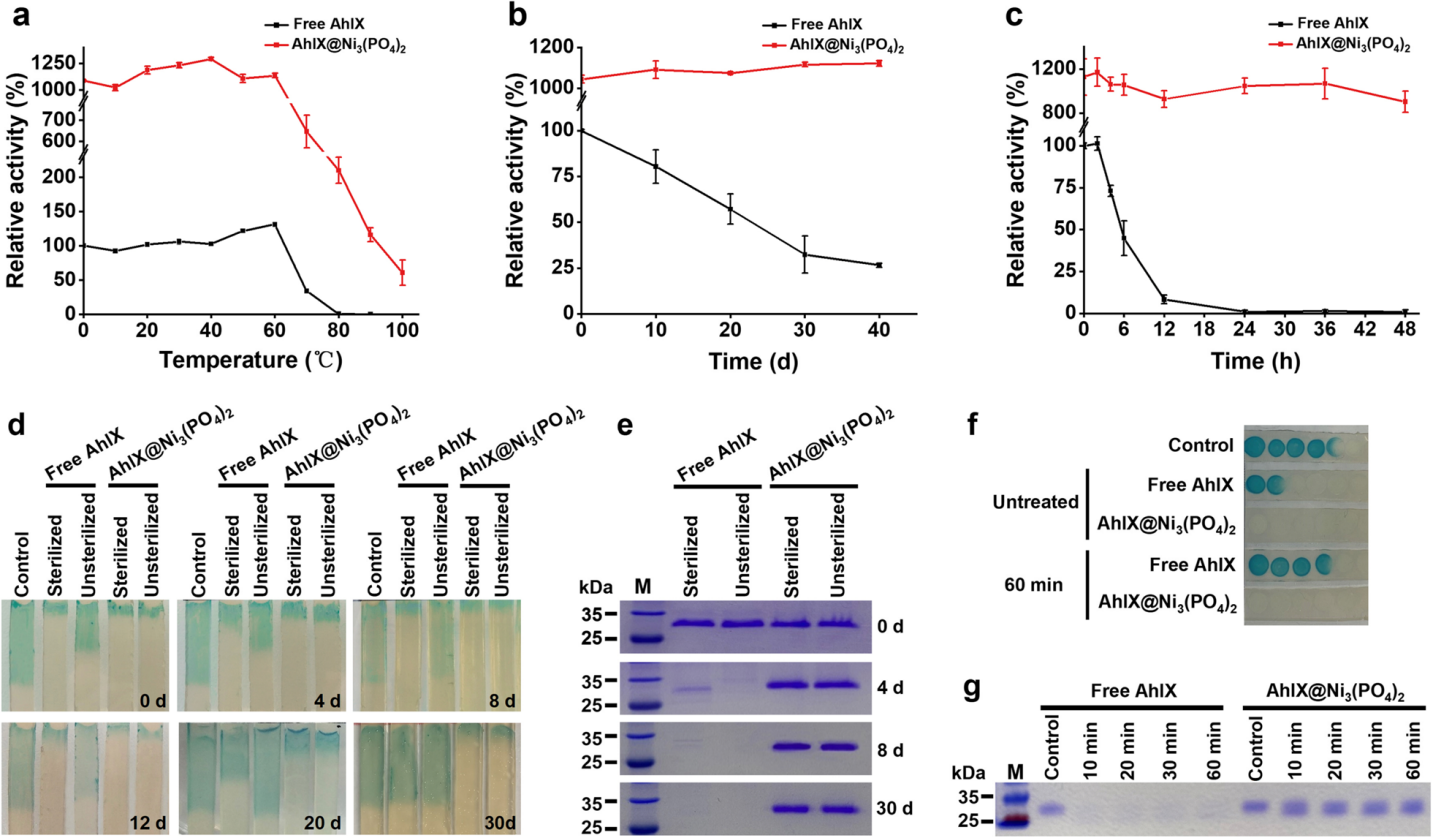
The stability of free AhlX and AhlX@Ni_3_(PO_4_)_2_ (AhlX concentration was 3.2 µM). **a** The thermal stability of free AhlX and AhlX@Ni_3_(PO_4_)_2_ at different temperatures for 30 min. The activity of free AhlX at 0 °C was defined as 100%. **b** The time-dependent thermal stability of free AhlX and AhlX@Ni_3_(PO_4_)_2_ at 25 ℃. The activity of free AhlX at day 0 was defined as 100%. **c** The time-dependent thermal stability of free AhlX and AhlX@Ni_3_(PO_4_)_2_ at 60℃. The activity of free AhlX at hour 0 was defined as 100%. **d** Stability of AhlX@Ni_3_(PO_4_)_2_ in the river water treatment. **e** Detection of the remaining AhlX by SDS–PAGE after the sterilized and unsterilized river water treatments. Standard marker protein (lane M). **f** Stability of AhlX@Ni_3_(PO_4_)_2_ in the proteinase K treatment. The control was 4 μM 3OC8-HSL. **g** Detection of the remaining AhlX by SDS–PAGE after the proteinase K treatment. Standard marker protein (lane M)

The stability of AhlX is crucial in practical applications. To investigate the stability of free AhlX and AhlX@Ni_3_(PO_4_)_2_, both were heat-treated at different temperatures for 30 min, and then the remaining activity was detected (Fig. 5a). Unlike free AhlX, which lost its total activity after a heat treatment for 30 min at 80 °C, AhlX@Ni_3_(PO_4_)_2_ retained 20.00% of its enzymatic activity after the same treatment, and the activity even remained at 5.00% after a heating treatment at 100 °C for 30 min. The RT stability of AhlX@Ni_3_(PO_4_)_2_ with a 3.2 µM AhlX concentration is better than that of free AhlX. After storage for 40 days, AhlX@Ni_3_(PO_4_)_2_ maintained full activity, while free AhlX retained only 26.52% of the activity (Fig. 5b). Consistent with this, AhlX@Ni_3_(PO_4_)_2_ had a better stability at 60 ℃ (Fig. 5c). AhlX@Ni_3_(PO_4_)_2_ maintained 80.26% of its activity after 48 h of heating at 60 °C, while the activity of free AhlX was completely lost after 24 h of heating at 60 °C. Overall, AhlX@Ni_3_(PO_4_)_2_ possesses better thermal stability than that of free AhlX. In a previous study [25], we found that AhlX displayed higher stability at a high concentration (0.6 mM) at 25 °C. However, the activity of AhlX showed a constantly declining trend when it was diluted to a working concentration of 3.2 µM (Fig. 5b). For practical applications, a low concentration of enzymes will contribute to a lower cost. Hence, AhlX@Ni_3_(PO_4_)_2_ shows better applicability as it has outstanding stability at lower concentrations. Meanwhile, compared with free AhlX, AhlX@Ni_3_(PO_4_)_2_ exhibited a better tolerance to organic solvents (Additional file 1: Figure S1). Free AhlX maintained an activity higher than 35% only when it was under 5% ethanol and acetonitrile. However, AhlX@Ni_3_(PO_4_)_2_ retained the same proportion of activity under 5% ethanol, acetonitrile, isopropanol, and *n*-butanol.

We speculated that the improvement in the stability of AhlX@Ni_3_(PO_4_)_2_ to temperature and organic solvent environments is due to the packaging of the enzymes being good. The stability of inorganic Ni_3_(PO_4_)_2_ under heat and acid–base conditions provides a relatively stable microenvironment for the AhlX wrapped in it, making it more resistant than free AhlX to changes in the external environment. To further validate our hypothesis, the samples were diluted with sterilized and unsterilized river water for different time spans. The activity was detected by the agar strip method (the longer the blue agar strip, the lower the activity), and the remaining protein was detected by SDS–PAGE (Fig. 5d, e). The activity of free AhlX treated with unsterilized river water on day 0 was significantly lower than that of the other three groups and basically lost all activity after 8 days. SDS–PAGE results showed that there was no obvious degradation of AhlX at the beginning. It is likely that there were some substances in the unsterilized river water that inhibited the activity of free AhlX. Considering both the activity test and SDS–PAGE results, it could be observed that a small residual amount of free AhlX in sterilized river water that was incubated for 8 days still showed great activity, but eventually it was completely degraded and lost activity after 30 days. It seemed that bioactive factors such as proteases or microorganisms could degrade the AhlX protein. At the same time, some substances can inhibit the activity of AhlX but do not induce degradation. In contrast, the activity of AhlX@Ni_3_(PO_4_)_2_ was maintained well when it was treated in sterilized or unsterilized river water for 30 days. AhlX of AhlX@Ni_3_(PO_4_)_2_ was barely degraded after a treatment in sterilized or unsterilized river water for 30 days.

To further evaluate the anti-proteinase ability of AhlX@Ni_3_(PO_4_)_2_, free AhlX and AhlX@Ni_3_(PO_4_)_2_ were digested with proteinase K for 0–60 min before activity and SDS–PAGE assays. The results showed that AhlX@Ni_3_(PO_4_)_2_ maintained full activity after proteinase K digestion for 60 min, while free AhlX completely lost its activity (Fig. 5f). This difference was derived from the anti-proteinase K effect of AhlX@Ni_3_(PO_4_)_2_ (Fig. 5g). AhlX@Ni_3_(PO_4_)_2_ can protect AhlX from proteinase K digestion. In summary, AhlX@Ni_3_(PO_4_)_2_ was embedded in a safe three-dimensional structure. This structure provided a good protective barrier and structural support for AhlX, and it avoids inhibition and degradation, which is caused by many factors, and enables AhlX to keep a stable activity for a long time. Due to its structural rigidity and stability in the natural environment, AhlX@Ni_3_(PO_4_)_2_ has superb potential in the control of bacterial infections.

## The biological control effect of AhlX@Ni_3_(PO_4_)_2_ on bacterial infections

**Fig. 6:**
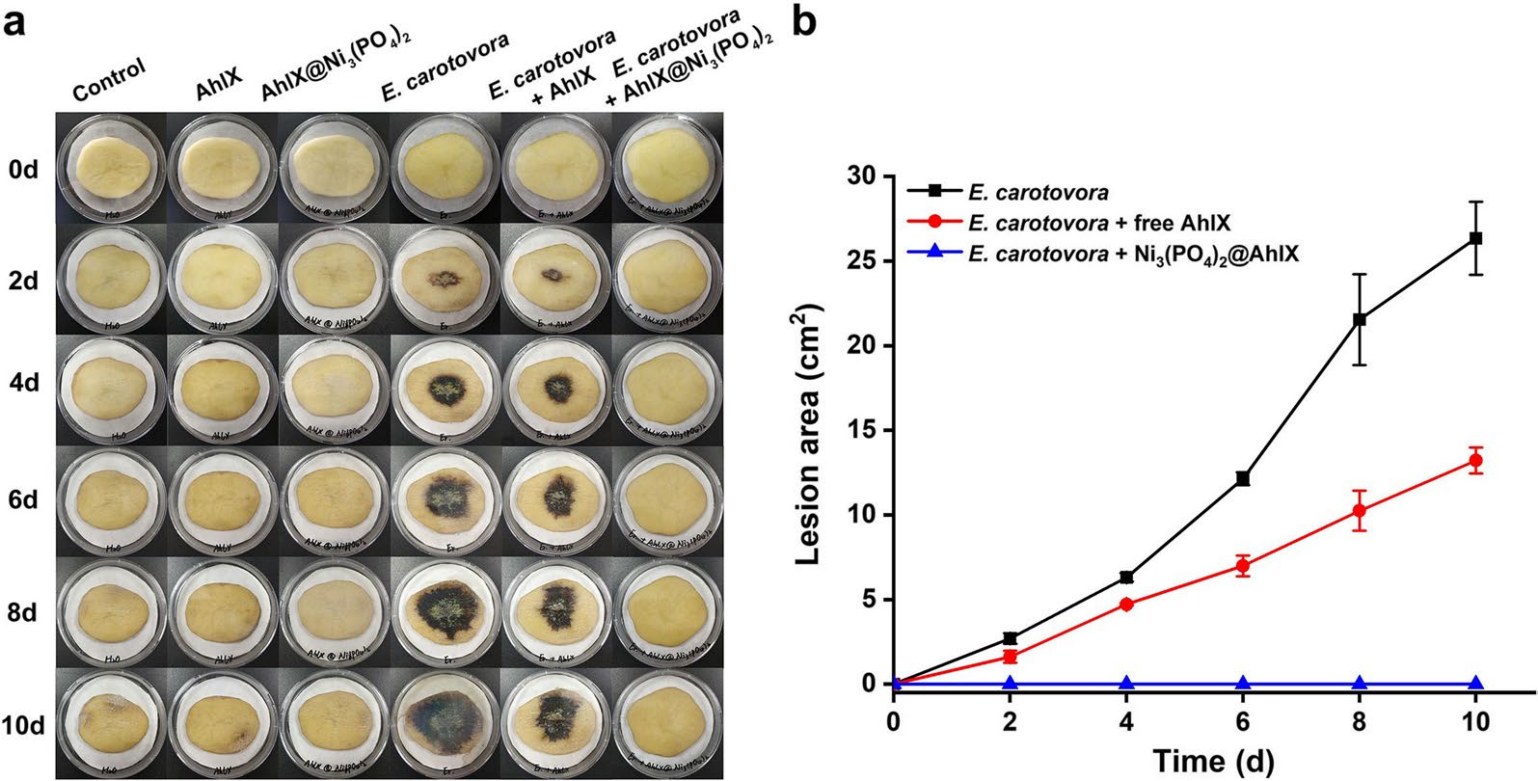
The biocontrol effect of AhlX@Ni_3_(PO_4_)_2_ on soft rot infectious disease caused by *E. carotovora*. **a** The biocontrol effect of AhlX@Ni_3_(PO_4_)_2_ on soft rot infectious disease caused by *E. carotovora* within 10 days. **b** The statistical results of soft rot development on potato slices that was caused by *E. carotovora* within 10 days

Many gram-negative plant pathogenic bacteria regulate virulence with AHL-based QS systems. With the goal of targeting QS systems, many biological control methods, including treatment with wild or transgenic QQ bacteria and transgenic plants expressing QQ enzymes, have been attempted against plant pathogens [11]. Compared with these methods, the directed use of highly stable enzymes in ecological environments has little effect on microecology and no gene contamination. In the in vitro biological control experiment, potato slices were treated with free AhlX and AhlX@Ni_3_(PO_4_)_2_, and these were all mixed with *E. carotovora* SCG1 (Fig. 6). The free AhlX treatment group showed that the potato soft rot caused by *E. carotovor* was effectively alleviated. In addition, AhlX@Ni_3_(PO_4_)_2_ completely inhibited potato soft rot. There were no soft rot symptoms on potato slices within 10 days of culture, and the inhibitory effect was as high as 100%. Compared with free AhlX, AhlX@Ni_3_(PO_4_)_2_ had an effective and lasting effect on the biological control of *E. carotovora* infection. The excellent biocontrol effect of AhlX@Ni_3_(PO_4_)_2_ is due to its efficient activity toward AHL molecules and strong tolerance to complex environmental factors.

**Fig. 7:**
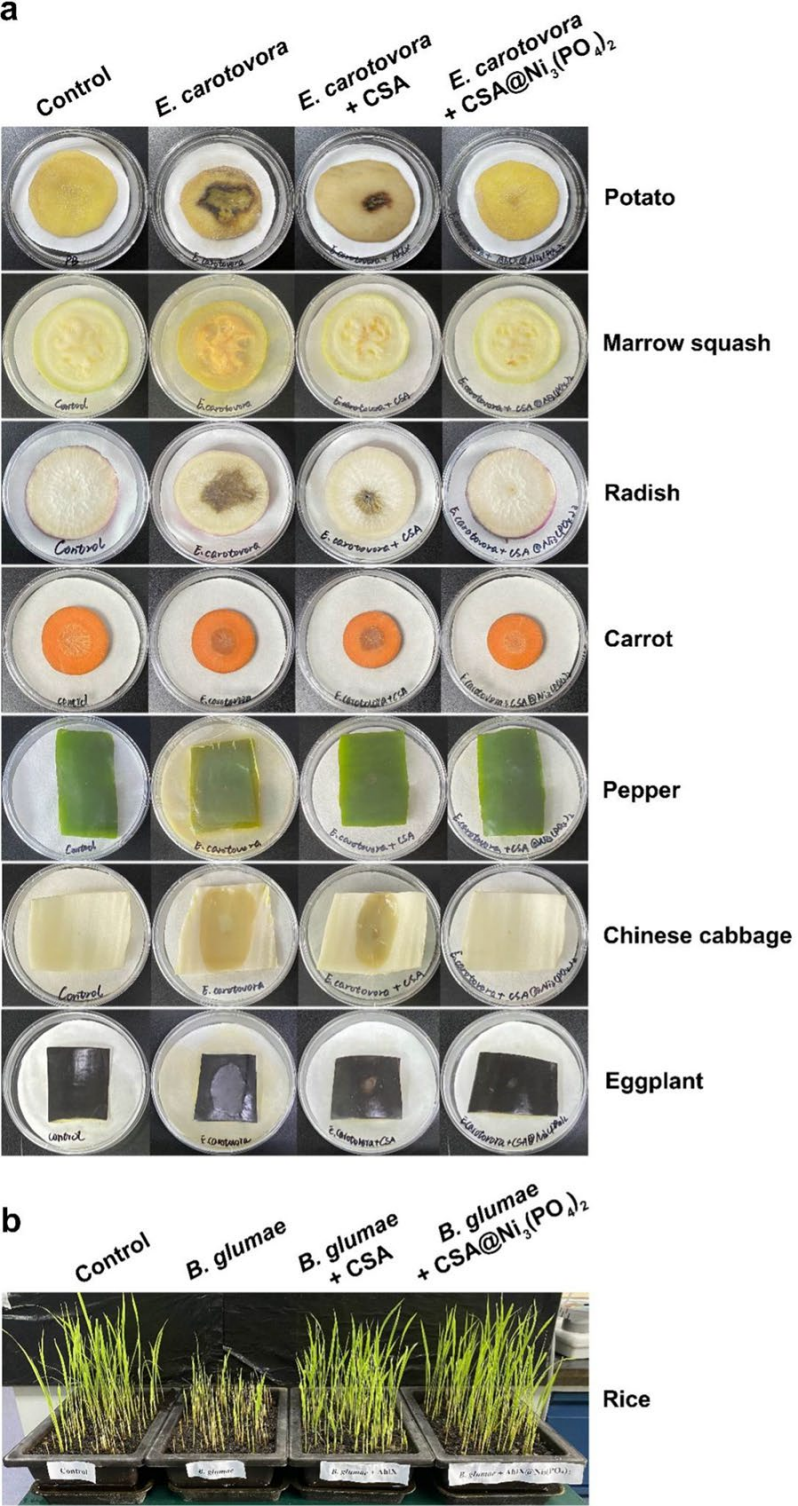
The inhibitory effect of CSA@Ni_3_(PO_4_)_2_ toward bacterial infections. **a** The in vitro inhibitory effect of CSA@Ni_3_(PO_4_)_2_ toward the soft rot caused by *E. carotovora* SCG1. **b** The in vivo inhibitory effect of CSA@Ni_3_(PO_4_)_2_ on rice seedling rot caused by *B. glumae*

For practical application, it is important not only to have a good biological control effect but also to produce agents in a convenient and inexpensive way. David et al. encapsulated an AHLase, PPH, in nanospherical capsules composed of tertbutoxycarbonyl-Phe-Phe-OH peptide [24]. It showed significantly improved thermal resistance and effectively attenuated *E. amylovora* infection in plants in the field compared to that of previous AHLases. However, the use of purified AHLases and expensive peptides still restrict its practical application due to the complex process and high cost. Using purified AhlX to produce EHNFs is also costly in scale-up production. To reduce the cost and streamline production processes, after cell lysis, the heat-treated crude supernatant of AhlX (CSA) was used to synthesize CSA@Ni_3_(PO_4_)_2_. We tested the in vitro inhibitory effect of CSA@Ni_3_(PO_4_)_2_ against *E. carotovora* infections. As shown in Fig. 7a, CSA effectively inhibited the *E. carotovora*-induced infectious symptoms of soft rot on potato, marrow squash, radish, pepper, Chinese cabbage and eggplant slices after incubation at 30 °C for different times, while full inhibition was observed on almost all of the plants inoculated with *E. carotovora* and CSA@Ni_3_(PO_4_)_2_ except for the marrow squash group, in which minimal soft rot was found. In addition, we validated the in vivo inhibitory effect of CSA@Ni_3_(PO_4_)_2_ against rice seedling rot that was caused by *B. glumae*. Inoculation with *B. glumae* alone resulted in serious seedling rot symptoms, whereas no seedling rot was observed in the rice seedlings treated with *B. glumae* and CSA@Ni_3_(PO_4_)_2_, and weaker seedling rot was detected in the rice seedlings treated with *B. glumae* and CSA (Fig. 7b). These data together illustrate that AhlX@Ni_3_(PO_4_)_2_ confers a great utility to AhlX and hold immense potential to become a next-generation antimicrobial agent.

## Conclusions

In this work, we prepared and characterized an extremely stable and highly active AHLase EHNF, AhlX@Ni_3_(PO_4_)_2_. AhlX@Ni_3_(PO_4_)_2_ maintained highly effective and durable activity in a complicated ecological environment. The efficient control of *E. carotovora* and *B. glumae* by AhlX@Ni_3_(PO_4_)_2_ in vitro and in vivo indicates its significant potential for further applications in controlling bacterial diseases.

EHNFs show apparent advantages when compared with some other biocontrol methods. First, in contrast to transgenic plants, there is no rigorous approval process [69]. Second, unlike transgenic biocontrol bacteria, there is no gene contamination with the environment [70]. Third, the materials used in the process of synthesizing EHNFs are all natural. Unlike enzymes immobilized with resin, AhlX@Ni_3_(PO_4_)_2_ can be easily degraded into natural substances. Finally, since the EHNF prepared from CSA possessed outstanding biocontrol capabilities, the preparation procedure is straightforward and can be easily scaled up at a low cost.

This is the first time that EHNF was used in the biological control of plant diseases. Practically, AHLases can also be applied in other fields. Many AHLases (AiiA, AiiM, *Sso*Pox, MomL and BpiB09) could effectively decrease the production of motility, virulence factors and biofilm formation of a common human and animal pathogen, *Pseudomonas aeruginosa*, in various in vitro or in vivo models, *Caenorhabditis elegans*, *Drosophila melanogaster* and rodents [13]. AiiA reduce the pathogenic of *Vibrio sp.* in brine shrimp and manila clam [71]. A membrane bioreactor with QQ bacteria entrapping beads successfully achieved the control of membrane biofouling by suppressing the AHLs responsible for motility, the secretion of extracellular polymeric substances and biofilm attachment to the membrane surface [72, 73]. Due to the high activity and stability of AhlX@Ni_3_(PO_4_)_2_, it has great promise for application in human and animal pathogens and biofouling control, and this hypothesis needs further experimental validation. In addition, although many AHLases have been isolated and studied in the laboratory, they cannot be applied practically due to their vulnerability in real environments. This work provides a feasible and universal strategy to improve the biological robustness of AHLases. Hence, AHLase-EHNFs are expected to solve the bottlenecks of poor stability and limited environmental tolerance, which have existed over two decades since AHLases were discovered, in practical applications.

## Supplementary Information

**Additional file 1:** **Figure S1.** The organic solvent tolerance of free AhlX and AhlX@Ni_3_(PO_4_)_2_. **Figure S2.** Detection of the remaining AhlX by SDS–PAGE after the sterilized and unsterilized river water treatments for 0 day. **Figure S3.** Detection of the remaining AhlX by SDS–PAGE after the sterilized and unsterilized river water treatments for 4 days. **Figure S4.** Detection of the remaining AhlX by SDS–PAGE after the sterilized and unsterilized river water treatments for 8 days. **Figure S5.** Detection of the remaining AhlX by SDS–PAGE after the sterilized and unsterilized river water treatments for 30 days. **Figure S6.** Detection of the remaining AhlX by SDS–PAGE after the proteinase K treatment. Standard marker protein (lane M).

## Data Availability

All data generated or analyzed during this are included in this published article.
