# Supplementary material for: N-acylhomoserine lactonase-based hybrid nanoflowers: a novel and practical strategy to control plant bacterial diseases
Source: J Nanobiotechnology. 2022 Jul 26;20:347. doi: 10.1186/s12951-022-01557-9 (PMC9327166; doi:10.1186/s12951-022-01557-9)
Supplement: Supplementary file 1 — Additional file 1: Figure S1. The organic solvent tolerance of free AhlX and AhlX@Ni3(PO4)2. Figure S2. Detection of the remaining AhlX by SDS–PAGE after the sterilized and unsterilized river water treatments for 0 day. Figure S3. Detection of the remaining AhlX by SDS–PAGE after the sterilized and unsterilized river water treatments for 4 days. Figure S4. Detection of the remaining AhlX by SDS–PAGE after the sterilized and unsterilized river water treatments for 8 days. Figure S5. Detection of the remaining AhlX by SDS–PAGE after the sterilized and unsterilized river water treatments for 30 days. Figure S6. Detection of the remaining AhlX by SDS–PAGE after the proteinase K treatment. Standard marker protein (lane M). [file 12951_2022_1557_MOESM1_ESM.docx]

**Additional file 1**

**AhlX-based hybrid nanoflowers: an effective strategy to quench plant bacterial diseases**

Yan Chen, Peng-Fu Liu, Jie-Qun Wu, Wan-Qing Yan, Sai-Xue Xie, Xuan-Rong Sun, Bang-Ce Ye*, and Xiao-He Chu*


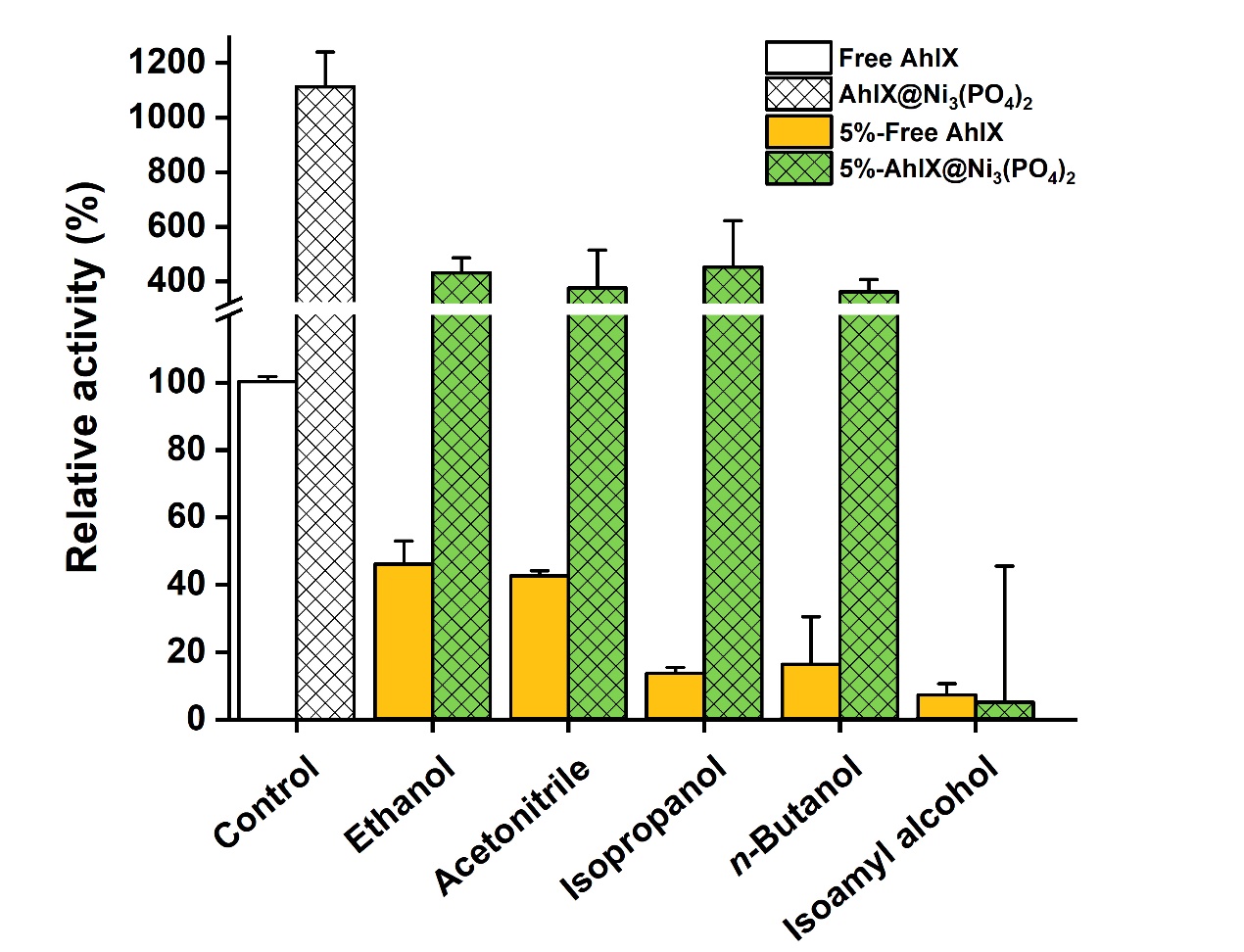


**Figure S1.** **The organic solvent tolerance of free AhlX and AhlX@Ni_3_(PO_4_)_2_.**


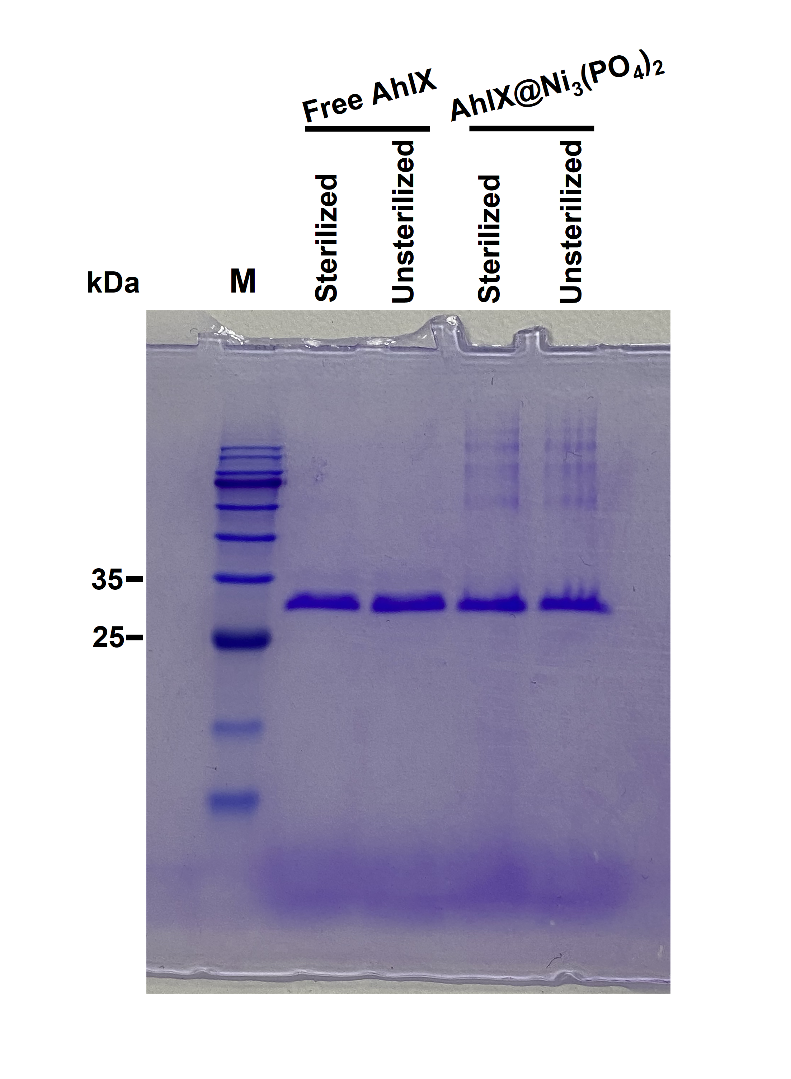


**Figure S2.** **Detection of the remaining AhlX by SDS–PAGE after the sterilized and unsterilized river water treatments for 0 day.**


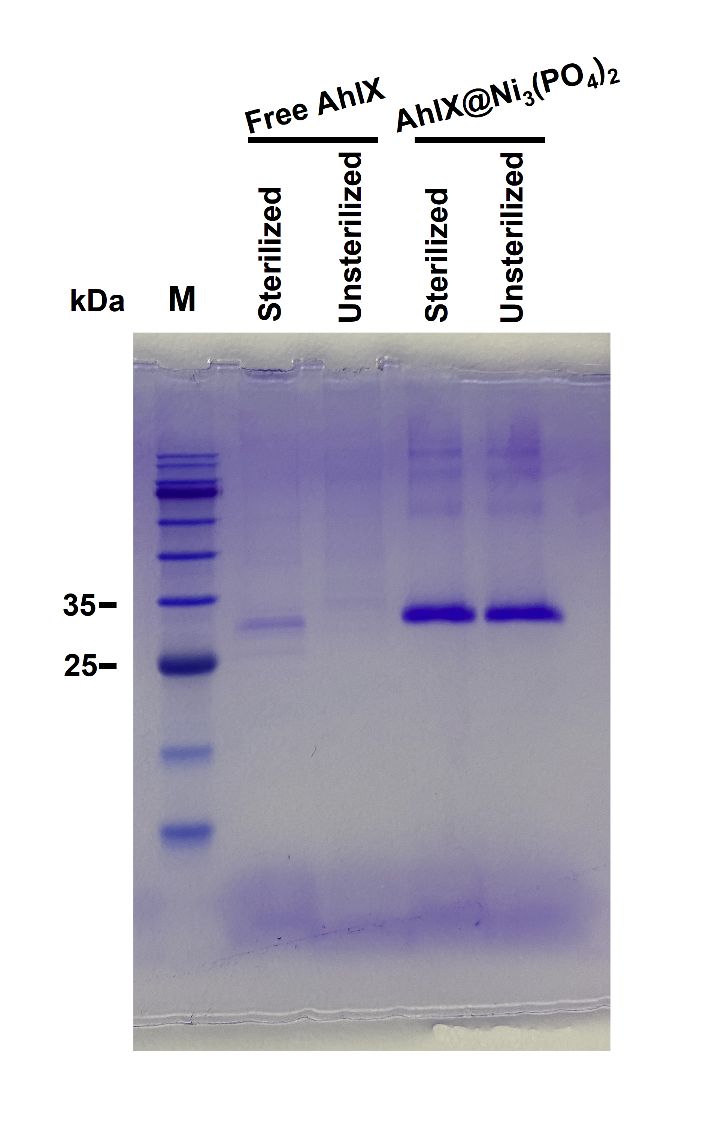


**Figure S3.** **Detection of the remaining AhlX by SDS–PAGE after the sterilized and unsterilized river water treatments for 4 days.**


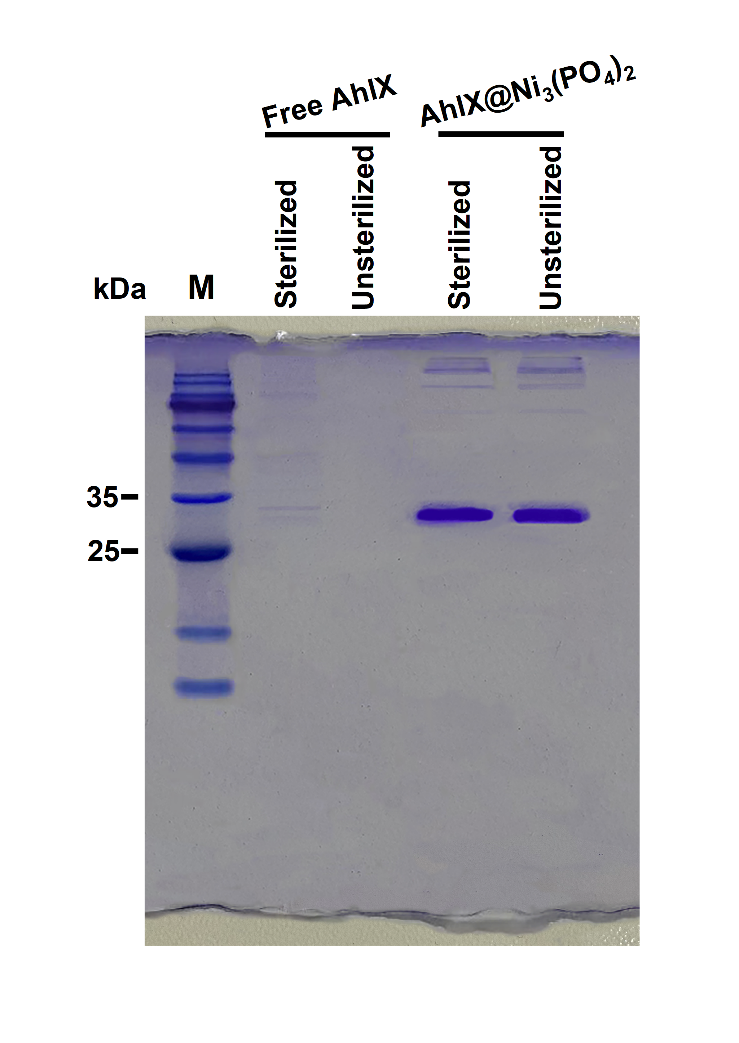


**Figure S4.** **Detection of the remaining AhlX by SDS–PAGE after the sterilized and unsterilized river water treatments for 8 days.**


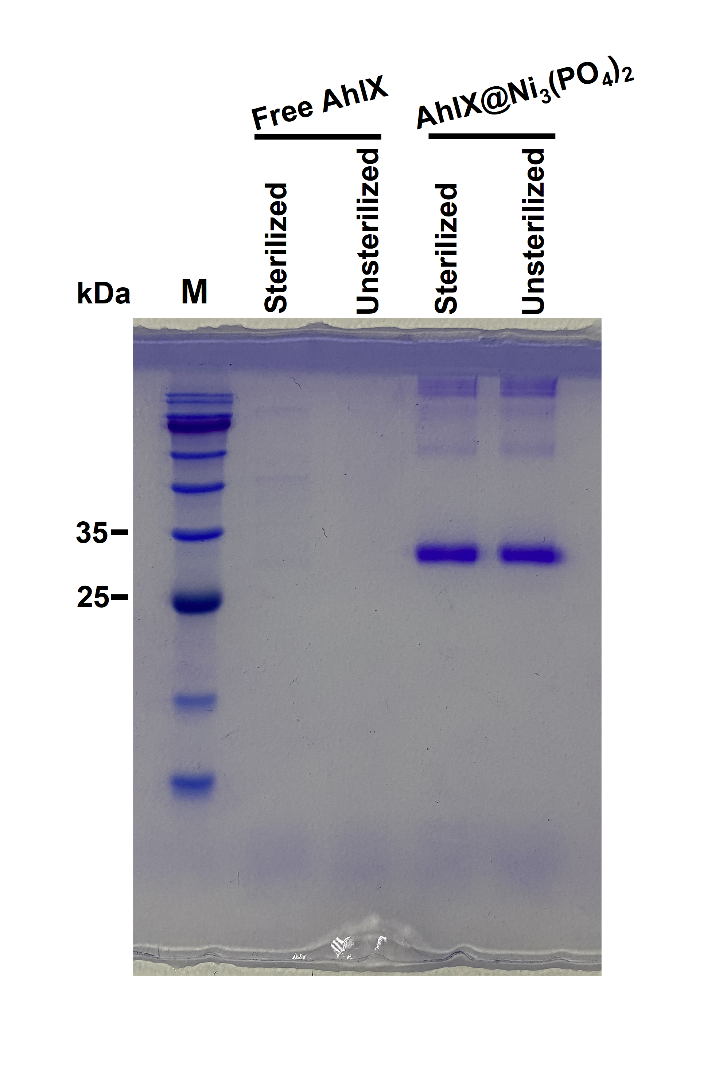


**Figure S5.** **Detection of the remaining AhlX by SDS–PAGE after the sterilized and unsterilized river water treatments for 30 days.**

**
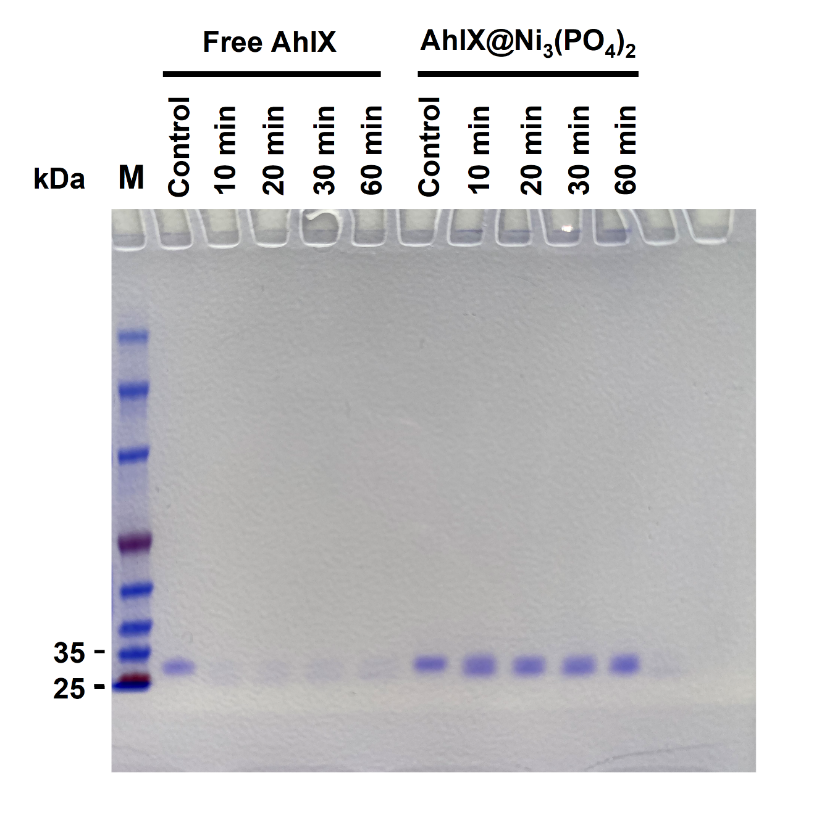
**

**Figure S6.** **Detection of the remaining AhlX by SDS–PAGE after the proteinase K treatment. Standard marker protein (lane M)**
